# National Survey of Morbidity and Risk Factors (EMENO): Protocol for a Health Examination Survey Representative of the Adult Greek Population

**DOI:** 10.2196/10997

**Published:** 2019-02-04

**Authors:** Giota Touloumi, Anna Karakatsani, Argiro Karakosta, Eleni Sofianopoulou, Panagiotis Koustenis, Magda Gavana, Yannis Alamanos, Maria Kantzanou, George Konstantakopoulos, Xenia Chryssochoou, Alexis Benos, Apostolos Vantarakis, Christos Hadjichristodoulou, Gregory Chlouverakis, Gregory Trypsianis, Paraskevi V Voulgari, Konstantinos Makrilakis, Stavros Liatis, George Stergiou, EMENO Study Group

**Affiliations:** 1 Department of Hygiene, Epidemiology and Medical Statistics Medical School National and Kapodistrian University of Athens Athens Greece; 2 Second Pulmonary Department, “Attikon” University Hospital Medical School University of Athens Athens Greece; 3 Department of Public Health and Primary Care School of Clinical Medicine University of Cambridge Cambridge United Kingdom; 4 Department of Political Science and History School of Political Science Panteion University of Social and Political Science Athens Greece; 5 Department of Primary Health Care, General Practice and Health Services Research Medical School Aristotle University Thessaloniki Greece; 6 Institute of Epidemiology, Preventive Medicine and Public Health Corfu Greece; 7 First Department of Psychiatry, Eginition Hospital Medical School National and Kapodistrian University of Athens Athens Greece; 8 Department of Psychology Panteion University of Political and Social Sciences Athens Greece; 9 Environmental Microbiology Unit, Department of Public Health Medical School University of Patras Patra Greece; 10 Department of Hygiene and Epidemiology Medical Faculty University of Thessaly Larisa Greece; 11 Laboratory of Biostatistics School of Medicine University of Crete Crete Greece; 12 Laboratory of Medical Statistics Medical School Democritus University of Thrace Thrace Greece; 13 Rheumatology Clinic, Department of Internal Medicine Medical School University of Ioannina Ioannina Greece; 14 Hellenic Diabetes Association Athens Greece; 15 First Department of Propaedeutic Internal Medicine Medical School National and Kapodistrian University of Athens Athens Greece; 16 Hypertension Center STRIDE-7, Third Department of Medicine, Sotiria Hospital School of Medicine National and Kapodistrian University of Athens Athens Greece

**Keywords:** health survey, chronic diseases, cardiovascular diseases, respiratory, risk factors, epidemiology, Greece

## Abstract

**Background:**

Main causes of death in Greece are cardiovascular diseases (CVDs), malignant neoplasms, respiratory diseases, and road traffic crashes. To assess the population health status, monitor health systems, and adjust policies, national population-based health surveys are recommended. The previous health surveys that were conducted in Greece were restricted to specific regions or high-risk groups.

**Objective:**

This paper presents the design and methods of the Greek Health Examination Survey EMENO (National Survey of Morbidity and Risk Factors). The primary objectives are to describe morbidity (focusing on CVD, respiratory diseases, and diabetes), related risk factors, as well as health care and preventive measures utility patterns in a random sample of adults living in Greece.

**Methods:**

The sample was selected by applying multistage stratified random sampling on 2011 Census. Trained interviewers and physicians made home visits. Standardized questionnaires were administered; physical examination, anthropometric and blood pressure measurements, and spirometry were performed. Blood samples were collected for lipid profile, glucose, glycated hemoglobin, and transaminases measurements. The survey was conducted from May 2013 until June 2016.

**Results:**

In total, 6006 individuals were recruited (response rate 72%). Of these, 4827 participated in at least one physical examination, 4446 had blood tests, and 3622 spirometry, whereas 3580 provided consent for using stored samples for future research (3528 including DNA studies). Statistical analysis has started, and first results are expected to be submitted for publication by the end of 2018.

**Conclusions:**

EMENO comprises a unique health data resource and a bio-resource in a Mediterranean population. Its results will provide valid estimates of morbidity and risk factors’ prevalence (overall and in specific subdomains) and health care and preventive measures usage in Greece, necessary for an evidence-based strategy planning of health policies and preventive activities.

**International Registered Report Identifier (IRRID):**

DERR1-10.2196/10997

## Introduction

### Background

Noncommunicable diseases account for 68% of all deaths worldwide [1], with cardiovascular diseases (CVDs), cancers, chronic respiratory diseases, and diabetes being among the top 10 leading causes. According to the World Health Organization [2], cardiovascular events were the world’s leading causes of death in 2015 (approximately 26.6% of deaths). In addition, about 14.4% of deaths worldwide are because of respiratory diseases [2].

Apart from age, gender and, family history, modifiable risk factors and their interactions account for about 60% of the CVD deaths. The main modifiable CVD risk factors are (1) behavioral factors such as unhealthy diet, sedentary lifestyle, smoking, and alcohol abuse; (2) cardiometabolic factors such as hypertension, hypercholesterolemia, diabetes, and increased body mass index; and (3) exposure to environmental pollutants and socioeconomic risk factors [2-6]. In addition, mental disorders, such as depression and anxiety, account for around 6.2% of the total disease burden [1], affecting approximately 10% of the population [7] and have also been associated with increased cardiovascular risk [8,9]. Over the past 30 years, following national preventive programs, age-standardized mortality rates have fallen in most countries, albeit to different degrees [10]. However, during the same period, initial increases followed by relatively small reductions in the incidence of CVD were observed in Eastern European countries as well as in Greece [10]. It is assumed that adoption of a modern lifestyle increased the prevalence of several CVD risk factors that contributed to the rising mortality rates.

In Greece, according to data from the Hellenic Statistical Authority [11], 50% of persons aged 15 years and over suffer from a chronic disease (25.2% increase compared with 2009). The financial crisis and austerity policies implemented in Greece in 2009 have various detrimental consequences on Greeks’ daily lives as well as on their health [12], including increase in heart attacks (50.0%), strokes (23.5%), and depression (80.8%), related also to rise in unemployment [11,13].

To design and implement prevention strategies for chronic diseases, national population representative baseline data on their prevalence are necessary. Nationwide health examination surveys (HES) combining information collected by interviews with participants’ physical examination consist the gold standard method to provide such data. HES have a long history in the United States [14,15] and since the 1990s, they have been widely introduced in Europe [16,17]. Setting up and implementation of a nationwide HES entail several difficulties and challenges, some of which are country or region specific.

In Greece, until recently, no such HES had ever been performed at the national level to record the prevalence of frequent chronic diseases and risk factors in a large, randomly selected sample, representative of the general population. Several health surveys have been conducted, which, however, were either restricted to specific regions or to high-risk groups [18-20] or were conducted within focused European projects [21,22].

### Objectives

Valid estimates of population morbidity and risk factor patterns, use of preventive measures, and health services and barriers in accessing health care are necessary to plan and implement effective prevention programs; however, such data are not available in Greece. The National Survey of Morbidity and Risk Factors (EMENO), a population-based health survey, was set up not only focusing on cardiovascular and respiratory diseases and related risk factors but also on assessing population well-being and use of health services, medicines, and preventive measures in a randomly selected sample of adults living in Greece. Combining health data with health examinations and blood sample testing and collection for future use, EMENO constitutes a unique health information tank and bio-resource. This paper presents the design and methodology of EMENO and discusses the challenges associated with organizing and implementing epidemiological HES using the *door-to-door* approach in a large representative sample.

## Methods

### Study Design

EMENO is a cross-sectional health examination survey combining health data collected by trained interviewers using standardized questionnaires and medical examinations conducted by trained physicians in a randomly selected sample of all adult people (aged ≥18 years) living in Greece, excluding those in supervised care or custody in institutional settings. It was funded by the European Union structural funds and National resources, coordinated by the Department of Hygiene, Epidemiology, and Medical Statistics of the National and Kapodistrian University of Athens Medical School, and implemented in cooperation with all the other Greek Medical Schools and the Institute of Epidemiology, Preventive Medicine, and Public Health. EMENO was initiated in May 2013 and completed in June 2016. The survey was delivered by *door-to-door* interviews using a computer-assisted personal interview (CAPI), augmented with data and physical examinations collected by trained physicians at scheduled appointments. Study physicians visited interviewed participants at their homes. In some small rural areas, interviewed participants were invited to visit local health care units where study physicians examined them. The *door-to-door* approach is common in health screening surveys, and it has been applied in some large scale, well-established surveys such as the National Health and Nutrition Exam Survey (NHANES) [23].

### Sampling Strategy

As a centralized person registry does not exist in Greece, the 2011 census constituted the sampling frame. Multistage stratified random sampling [24,25] based on 2011 Census was applied to select the sample. The sampling procedure consisted of 4 stages: (1) initial sampling units (specific regions), (2) building blocks, (3) households, and (4) individuals.

Stage 1: The whole country was stratified by geographical region (9 regions plus the Greater Attica Area and the Greater Thessaloniki Area) and by degree of urbanization (urban: ≥10,000, semi-urban: 2000-9999, and rural: up to 1999 inhabitants) resulting in 33 strata. As Greater Athens and Thessaloniki Areas account for approximately 45.62% (4,937,936/10,823,686) of the total population, and to increase the precision of estimates for the geographical region subdomain, sampling fraction was allowed to differ by stratum, over-representing smaller strata, and under-representing larger strata, provided that the minimum sampling fraction per region (0.042% in Attica) does not get more than treble the maximum one (0.116% in Ionian islands). Table 1 displays the sampling fraction per stratum.

**Figure 1:**
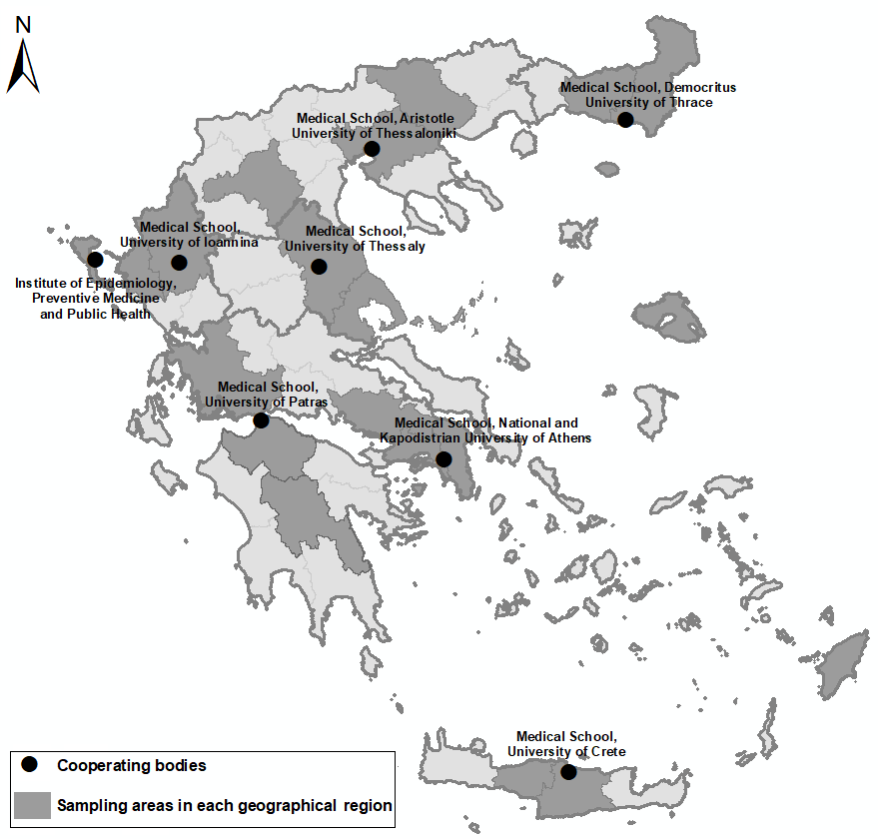
Final sample mapping.

Given that it was not possible to visit each prefecture in each geographical area, apart from Ionian Islands’ region where 1 island (Corfu) was selected, in all other geographical regions, 2 prefectures were randomly selected (Table 1 and Figure 1). Greater Athens area was further divided into 4 regions: Athens, East Attica, West Attica, and Piraeus. Thus, the total primary sampling units (PSUs) were 66 (22 regions multiplied by 3 degrees of urbanization).

Stage 2: Within each PSU, area segments comprising census blocks or combination of blocks were randomly selected (sampling points). From each sampling point, 12 households in urban and semi-urban and 8 in rural areas should be available for interview. Assuming a 50% response rate, the needed number of households per sampling point was 18 for urban and semi-urban and 12 for rural areas. The target sample size was 6000 adults (1 per household). On the basis of the population size of each PSU and considering the corresponding sampling fraction as well as the average number of households per PSU, the total number of sampling points per PSU was determined. In total, 577 sampling points were selected (295 in urban, 89 in semi-urban, and 193 in rural areas). Hellenic Statistical Authority provided the coordinates (latitude and longitude) of each building block within each sampling point, and then these were transferred to maps through Geographic Information Systems to become available to the interviewers.

Stage 3: Within each sampling point, eligible households were selected via systematic sampling. All interviewers were provided with the maps of the selected sampling points. Standardized operating procedures (available on request) were developed on how to select the eligible households within each sampling point (eg, starting from the top left corner of the map, in blocks of flats starting from the top floor and moving clockwise, spiraling downwards). One household of every *x* households was selected, with the step x ranging from 4 (ie, leaving 4 households and selecting the fifth) to 2, depending on population density. Each map was accompanied by its corresponding step. Interviewers had to describe all followed steps in a specific database. Only residential houses were eligible. If there was no reply in the first attempt, interviewers had to visit the household 2 more times in different hours (eg, once in the morning and once in the evening and one time during weekends). If there was information that a house was not inhabited for a long period or that it was a vacation place, it was considered as noneligible. Similarly, if after 3 attempts nobody replied, the household was also considered as noneligible. If there was a reply but the inhabitants refused to receive information about the study, this was considered as nonresponse.

Stage 4: All eligible (ie, adults ≥18 years) individuals within a household were listed and 1 individual per household was randomly selected (the one who had a birthday last, also known as *most recent birthday* method [26]). If the selected individual agreed to participate, an appointment was arranged for interview. People who refused (counted as nonresponders) were substituted by responders from the next eligible household, until the target number of individuals per sampling point (12 or 8) was met. Response rate was estimated as the number of interviews over the number of eligible households reached.

**Table 1:** Sampling plan and response rates (N=10,823,686).

Area and urbinity		Distribution of the required sample (n=6000) by urbanity	Sampling fraction (%)	Response rate (%)
**Thrace (N=371,208)**		**n=303**		
	Urban	154	0.8157	65.8
	Semi-urban	66	0.8163	75.0
	Rural	83	0.8170	81.1
**Macedonia (N=1,290,404)**		**n=666**		
	Urban	257	0.5165	76.6
	Semi-urban	141	0.5179	82.2
	Rural	268	0.5149	77.2
**Thessaloniki (N=1,110,312)**		**n=585**		
	Urban	451	0.5263	50.8
	Semi-urban	106	0.5291	73.8
	Rural	28	0.5283	80.0
**Thessaly (N=734,754)**		**n=464**		
	Urban	240	0.6309	52.7
	Semi-urban	79	0.6317	71.7
	Rural	145	0.6321	82.9
**Central Greece (N=758,192)**		**n=479**		
	Urban	147	0.6317	75.0
	Semi-urban	139	0.6353	83.8
	Rural	193	0.6293	95.4
**Epirus (N=336,856)**		**n=291**		
	Urban	104	0.8621	45.2
	Semi-urban	58	0.8718	83.3
	Rural	129	0.8621	95.2
**Ionian (N=207,855)**		**n=242**		
	Urban	50	1.1696	47.2
	Semi-urban	54	1.1669	71.8
	Rural	138	1.1614	76.2
**Aegean (N=507,167)**		**n=355**		
	Urban	129	0.7013	78.1
	Semi-urban	98	0.7008	79.3
	Rural	128	0.6978	90.1
**Attica (N=3,827,624)**		**n=1615**		
	Urban	1529	0.4218	73.1
	Semi-urban	74	0.4252	61.8
	Rural	12	0.4299	88.9
**Peloponnese (N=1,049,347)**		**n=596**		
	Urban	238	0.5682	82.7
	Semi-urban	120	0.5688	89.5
	Rural	238	0.5672	98.4
**Crete (N=629,967)**		**n=404**		
	Urban	192	0.6406	82.9
	Semi-urban	89	0.6456	70.6
	Rural	123	0.6398	68.1

### Sample Size

On the basis of the ATTICA study results [27], the overall prevalence of hypercholesterolemia (total serum cholesterol levels >200 mg/dL or use of lipid lowering agents), hypertension (average blood pressure levels >140/90 mmHg or receiving antihypertensive medication), and of diabetes mellitus (fasting blood glucose >125 mg/dL or receiving antidiabetic medication) was about 40%, 32%, and 7%, respectively. Calculating sampling errors using the corresponding formula for stratified sampling [25] and with a target sample size of 6000 individuals, the above-mentioned prevalence could be estimated with 1.28%, 1.22%, and 0.66% precision, respectively.

### Variables and Questionnaire Development

A scientific committee (SC), which included experts in epidemiology, medical statistics, internal medicine, hypertension, cardiology, respiratory medicine, and psychology, was formed to act as an expert panel tasked with reaching consensus on survey instrument development. For questionnaires’ forming, where possible, questionnaire items from existing survey instruments were adapted. For that, a systematic literature review of international and Greek bibliography and a review of the large epidemiological studies’ websites (eg, NHANES; Survey of Health, Ageing and Retirement in Europe; and European Health Examination Survey) were conducted. New questions were drafted by the core research team and scientific committee. Two different questionnaires were formed for interviewers and doctors.

The final interviewers’ questionnaire was divided into the following sections: (1) sociodemographic data, including data for insurance coverage, (2) health status (general health, depression and anxiety screening, self-reported chronic diseases, subquestionnaires for coronary and peripheral artery disease, chronic pulmonary obstructive disease (COPD) and asthma, sleep, menstruation, and personal well-being), (3) health care system (use and satisfaction, health expenditures, medicines use, screening, and vaccinations), and (4) factors affecting health (self-reported height and weight, physical activity, adherence to Mediterranean diet, alcohol consumption, nutrition insecurity, smoking, environmental exposure, and household economic characteristics). The sources of the questionnaire items are shown in Multimedia Appendix 1. The total number of items was 142, but through skipping and filters, some respondents answered much fewer items. Although participants were interviewed by CAPI, there were self-administered laminated cards either to most sensitive questions (eg, psychological well-being, health care system, and self-reported chronic diseases) or to questions required visual presentation for a better understanding (to indicate the exact point of pain in chest in the coronary heart disease sub questionnaire or leg pain in the arteriopathy questionnaire). The vast majority of the questions were *close ended*.

Physicians’ questionnaire included general information about physical examinations (consultation outcome, place, physical examinations, date, and duration) and sections according to the physical measurements and their exclusion criteria: (1) somatometry (height, weight, waist circumference, and left and right arm circumference); (2) blood pressure and presence of atrial fibrillation (the latter for those aged ≥65years); (3) spirometry; and (4) blood sampling. Collected blood samples were analyzed for total cholesterol, high-density lipoprotein, low-density lipoprotein, glucose, glycated hemoglobin, triglycerides, and transaminases. Standardized instruments (see Multimedia Appendix 2) were used across the country for physicians’ measurements, whereas standardized operating procedures (SOPs) were developed by the EMENO SC for each measurement, spirometry, and blood samples’ collection. A researchers’ manual was developed containing information about health surveys; sampling procedure; interview techniques; detailed guidelines on filling each questionnaire item; the SOPs for physicians’ measurements; description of the used instrument; and management of collected blood samples.

Survey questionnaires were tested and validated (time and clarity of questions) in 30 volunteers from urban, semi-urban, and rural areas and different ages. According to Sudman [28], “it usually takes no more than 12-25 cases to reveal the major difficulties and weaknesses in a pretest questionnaire” and “20-50 cases is usually sufficient to discover the major flows in a questionnaire.” All research teams participated in this phase. After questionnaires’ pretesting, additions and/or corrections were made, if necessary.

### Clinical and Biochemical Measurements

Detailed SOPs were developed, but here, we briefly summarize methodology for clinical and biochemical measurements. Height and weight were measured without shoes and in light clothes. Arterial blood pressure was measured in sitting position after at least 5 min of rest; 3 valid consecutive measurements were taken with 1-min interval between them. Spirometry was performed in a standardized manner in eligible individuals (sitting position after at least 15 min at rest; participants should not have received any inhaled medicine or smoke within 1 hour before spirometry and should not have consumed any food during the previous 30 min). Portable spirometers (Multimedia Appendix 2) compatible with American Thoracic Society and European Respiratory Society requirements were used [29]. In total, 8 hours fasting serum samples were collected for determining cholesterol, glucose, glycated hemoglobin, triglycerides, and transaminases levels. For glucose, serum samples were collected in special sodium fluoride tubes. Additional samples were stored for future research if consent was provided. Upon specific consents, additional plasma samples for future molecular analyses and whole blood samples for DNA extraction were collected.

### Ethics

EMENO study was approved by the Ethics and Deontology Committee of the National and Kapodistrian University of Athens (date: November 8, 2012, protocol: 1742) and by the Hellenic Data Protection Authority (date: December 7, 2012, protocol: ΓΝ/ΕΞ/1069-1/07-12-2012). A modified version of the informed consent form (ICF) was approved by the Ethics and Deontology Committee of the National and Kapodistrian University of Athens (date: March 6, 2013, protocol: 6315). All participants were given enough time to read carefully the ICF and to ask relevant questions before they signed it. Apart from the ICF for participating in the EMENO, separate ICFs were provided for storing leftover samples and for taking additional samples for research not including or including DNA analysis.

Barcodes were prepared with unique individual codes. These codes were a combination of digits demonstrating the region, the interviewer, and serial number of the participant. Barcodes were given to interviewers and physicians and attached to all forms and questionnaires as well as referral form for blood examinations and blood tubes. Specific deep freeze barcodes were printed and used to store aliquots till blood testing or preservation for future research (if relevant ICFs were signed). Personal data were stored in a separate safely stored file and were linked by code with the rest of the participants’ information. Access to personal data was limited to each region’s principal investigator and was only used to contact participants for sending thank your letters, the medical report, and for future follow-up, if they had consented to it.

### Researchers’ Training

Training materials were developed by the SC on data collection through questionnaire, sampling, blood collection, and physical measurements protocols. All training materials were available on electronic database, accessible by all researchers. A 2-days training program was organized on September 12, 2013, to September 13, 2013 and lasted 16 hours acknowledged by the Pan-Hellenic Medical Association. All selected (by the time of training implementation) field study staff (30 individuals) attended it. Training sessions included a practical session, where field researchers completed questionnaires in groups of 3 to 5 people and under the supervision of a member of the scientific committee, whereas physicians did medical examinations in volunteers. All participants completed a training program evaluation form. After the core training, a train-the-trainer approach was used to decentralize training in each region. The trained staff, under supervision of the principal investigator of each region, was in charge for training the new staff entering the group throughout the study implementation.

### Pilot Study

We piloted the whole study in a sample of 160 people from urban and rural areas in 7 regions of Greece (Athens, Thessaloniki, Thessaly, Peloponnese, Crete, Epirus, and Thrace). The main objectives or the pilot study were to (1) determine feasibility of the study protocol, (2) check adequacy of research tools, (3) test the performance of measurement instruments, (4) assess the effectiveness of the sampling approaching method, (5) identify potential logistical problems or deficiencies, (6) collect preliminary data for the survey, and (7) check the competence of investigators’ and physicians’ training.

Although field researchers followed the provided instructions and protocols (they had been provided with special identities and letters of information, whereas local police station was informed about the survey), the response rates were very low, ranging from 27% to 50%. However, among those who consented to EMENO, a particularly high percentage (ranged from 90%-100%) also participated in the physical examinations, indicating that the main difficulty was to gain the trust of eligible households to open the door. This was a function of field researchers lacking prior experience and of limited study promotion. The issue was extensively discussed by the SC and additional ways to effectively promote the study were identified (see Study Dissemination section). Experienced field researchers (mainly working in surveys run by the Hellenic Statistical Authority) were invited to provide additional guidance to EMENO researchers. Women’s participation was particularly high (3447, 57.50%), revealing the importance of strictly following the sampling method. Following pilot study’s data quality control, mistakes in the Web-based database (skip mistakenly) were detected and corrected, whereas further explanations were provided for specific questions (eg, what we mean by *intense physical exercise*). Further adjustments to the questionnaire were made wherever this was deemed necessary.

### Implementation of the Main Field Study

The principal investigator of each collaborating School of Medicine had the field study of the nearby regions under his or her responsibility (Figure 1). Before starting the filed study, researchers and physicians signed a confidentiality form and vaccination for hepatitis B was recommended. In addition, they were provided with study identities with their photo and personal information. In EMENO’s website, apart from information about the study, the photos, and key curriculum vitae information of the researchers were posted. Field researchers were provided with maps of each sampling point, a laptop with the database, and all necessary printed material. For obviation of participants’ mistrust, an informative letter was sent to the local police departments informing about the study. In addition, in case of blocks of flats, an official notification letter was given to the apartment manager to facilitate the entry of field researchers in the eligible flats.

Blood samples were kept in cold environment (at 4°C) until transported, latest within 12 to 18 hours, to collaborating local laboratories for centrifugation. Centrifugal aliquots were stored in the collaborating laboratories at −80 °C until they were sent to the central laboratory (National Retrovirus Reference Center, Laboratory of Hygiene, Epidemiology and Medical Statistics of the Medical School of the University of Athens) for testing.

The ideal blood sampling procedure was determined according to local circumstances: In urban and semi-urban areas, physicians made home visits at scheduled appointments. In rural areas, and in agreement with the participants, blood sampling and medical examinations were performed at nearby health centers.

### Study Dissemination

Study informational brochures were developed and distributed to eligible houses. Additional promotion actions included:

1. Informing meetings with local authorities (mayors and deputies of social policy). The study was conducted with the cooperation of local authorities. The collaboration included (1) an invitation to the study through a letter from the municipality. The researchers’ names were mentioned in the letter, (2) announcement of the study on municipality’s site, (3) information brochures distribution at infrastructures under the responsible person of the Municipality (such as elderly protection centers, social welfare, and pharmacies), (4) phone calls to eligible households, if these were available in the municipality structures, (5) studies’ posters suspension at key point areas of the municipality, and (6) escorting the field researcher by an employee of the Municipality, where feasible.
2. Request and approval of study information letter by the Ministry of education. The Ministry sent a letter of approval to all schools of primary and secondary education, so that promotion functions would be welcome.
3. Request and approval of a letter to promote the study by the Holy Synod. The Holy Synod sent a letter of approval to all orthodox churches of Greece, so that the congregation would be encouraged to participate in the survey.
4. Events in central locations adjacent to sampling points. These events were attended both by the scientific leaders of each region as well as the field researchers and volunteers (mainly students of medical schools).
5. Central and local press conferences in all study regions, where the study objectives and its expected benefits were presented.

### Sending Medical Results to the Participants

Each participant received the following for his or her participation: (1) appreciation letter, (2) results of the medical examinations, (3) medical report (with recommendations) based on the personal medical findings, (4) pyramid of the Mediterranean diet, and (5) National Organization for the Provision of Healthcare Services’ recommendations for preventive measures and adult vaccination. A network of specialized physicians was established to which participants identified with urgent previously undiagnosed conditions (eg, hypertension and COPD) were administered at no cost for the participant.

### Information System

The central database was hosted on a central server, located in the Laboratory of Hygiene, Epidemiology and Medical Statistics of the Medical School of the National and Kapodistrian University of Athens. Actions were taken for data security. All data related operations were made through secure transactions encrypted with 128-bit encryption, and both the databases were Atomicity Consistency Isolation Durability-compliant to ensure that all the previously described operations are unique, consistent, isolated, and durable. During field study, data quality assessments were conducted regularly, and adjustments were made when necessary.

### Statistical Analysis

EMENO has a complex study design, with varying selection probabilities across regions. Thus, for the statistical analysis, sampling weights, being the reciprocal of the selection probabilities, should be applied. Although analyses using the sampling weights should give representative estimates, departures in the age and sex distribution from the respective ones based on the 2011 census are expected as women and older individuals are usually over-represented in this kind of surveys. To adjust for such discrepancies, auxiliary information from the 2011 census to reflect Greek population’s distribution of age and gender by geographical region was used (poststratification weighting). Thus, the initial weights are multiplied by a correcting factor *f*_*PSi*_ as shown below:

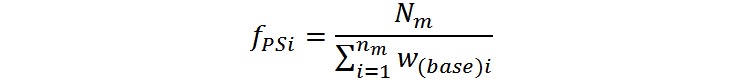



Where, *w*_*(base)i*_ is the sampling weight, *N*_*m*_ the population’s total in the poststratum, *m*_*i*_ and *n*_*m*_ is the corresponding sample’s size. Thus, the final weights are *w*_*PSi*_*=w*_*(base)i*_*f*_*Psi.*_

Adjustment for nonresponse could also be applied. In the EMENO study, additional data for nonresponders’ characteristics were not available. However, response rates were recorded within sampling points. Adjustment for nonresponse could be done by multiplying the initial weights with the reciprocal of the response rate under certain (mostly untested) assumptions. Investigation of the effect of adjusting for nonresponse on study estimates is being planned.

The weighting procedure may lead to weights with extreme values. In such cases, trimming of the weights (usually at the 95th or the 99th centile of their distribution) is suggested, as extreme weights may have a serious impact on the variance of the estimates. Untrimmed weights are adjusted so that the sum of the weights after trimming to be equal to the sum before trimming. Weights will be graphically investigated to assess the necessity of weight trimming. To determine estimates’ variances, we applied the Taylor series linearization method as recommended by National Center for Health Statistics and also used in the NHANES study [30].

Some EMENO participants refused to have physical examinations and/or to provide blood samples. However, those who provided blood samples or had physical exams may differ from those who did not. To adjust for that, we can use either inverse probability weighting or multiple imputation methods. In a preliminary sensitivity analysis we performed [31], we found that these 2 methods provide comparable results; however, this issue needs further investigation.

## Results

### Response Rates

**Figure 2:**
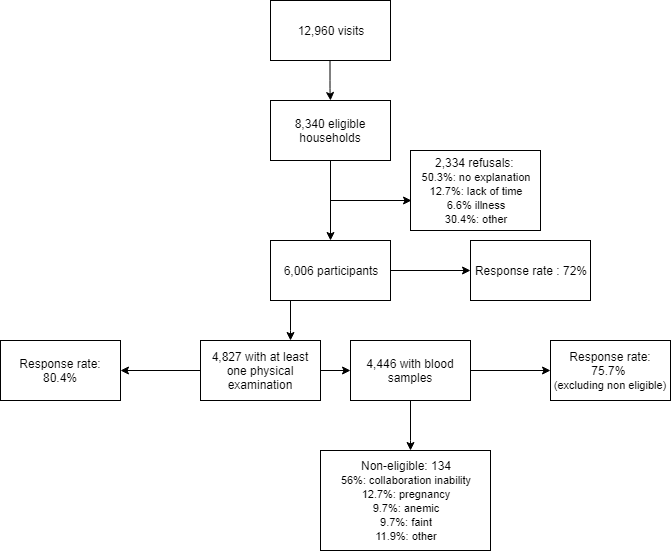
Sampling flow chart.

The sampling flowchart is shown in Figure 2. In total, 12,960 households were visited, of which 4620 were noneligible (716 professional use, 1132 uninhabited houses, 1882 no reply at 3 attempts, 372 vacation homes, 365 away from home for more than 2 weeks, and 153 undefined). Among eligible households, the overall response rate for interviewing was 72%. Response rates differed substantially by geographical region and degree of urbanization, being higher in small or rural places and lower in big cities (Table 1). Among interviewed participants, 80.54% (4827) participated in at least one physical examination, 74.20% (4446) had blood tests, and 60.44% (3622) spirometry. In total, 3580 individuals provided signed ICF to use leftover or additionally collected samples for future research, of whom 3528 also allowed for DNA extraction. The number of available samples by sample type were: 3259 serum; 3410 plasma and 3113 whole blood.

### Demographic Characteristics of the Participants

In total, 13 out of the 6006 EMENO participants refused to provide their age. As age is necessary to compute poststratification weights, all results provided are restricted to the 5993 participants with available age. In Table 2, the age and sex distribution of the study population as well as of the reference population are shown. Compared with the reference population (ie, people living in Greece based on the 2011 census), women and older people were over-represented in the sample. However, applying poststratification weighting resolved these discrepancies. Sociodemographic characteristics after poststratification weighting are shown in Table 3. The mean (SD) age of the population was 49.3 (18.6) years; 51.5% (3447) were women; and 46.7% (2597) had graduated secondary or postsecondary school. Over half (3936, 61.0%) were married or in cohabitation. About 12.6% (666) were born in a country other than Greece. Data were gathered during the economic crisis in Greece (2013-2016) and this is reflected in the family income; household monthly income was up to 900€ for 39.8% (2401) of the population, whereas 15.4% (784) were unemployed. The estimated (95% CI) unemployment rate among those aged <65 years was 28.7% (26.9-30.5). 

Further statistical analysis of the data is underway, and first results are expected to be submitted by the end of 2018. Main results will concern cardiovascular and respiratory diseases and their corresponding risk factors’ prevalence among adult Greek population, assessing the degree of implementation of recommended prevention measures and possible barriers to access to the public health system as well as socioeconomic factors affecting health. In addition, mapping of air pollution levels and investigation of their impact on citizens’ health will also be conducted.

**Table 2:** Age and sex distribution in the National Survey of Morbidity and Risk Factors (EMENO) sample and the corresponding distributions based on the 2011 census.

Age (years)	Population (Census 2011), n (%)		Sample (EMENO), n (%)	
	Male	Female	Male	Female
18-29	817,789 (9.16)	765,498 (8.57)	298 (4.97)	336 (5.61)
30-39	827,542 (9.27)	807,762 (9.05)	350 (5.84)	469 (7.82)
40-49	781,112 (8.75)	799,983 (8.96)	406 (6.77)	614 (10.24)
50-59	677,018 (7.58)	714,836 (8.01)	425 (7.09)	688 (11.48)
60-69	543,421 (6.09)	590,624 (6.62)	484 (8.08)	627 (10.46)
70-79	456,247 9 (5.11)	560,995 (6.28)	383 (6.39)	474 (7.87)
80+	231,746 (2.60)	351,588 (3.94)	200 (3.34)	241 (4.02)
Total	4,334,875 (48.56)	4,591,286 (51.44)	2546 (42.48)	3447 (57.52)

**Table 3:** Demographic characteristics of study participants after poststratification weighting (N=5993).

Demographic characteristics		Statistics
**Gender, n (weighted %)**		
	Male	2546 (48.5)
	Female	3447 (51.5)
**Age (years)**		
	Weighted median (interquartile range)	47.7 (34-64)
**Educational level, n (weighted %)**		
	Up to primary	2114 (28.8)
	Up to secondary or postsecondary	2575 (46.7)
	University or higher	1200 (23.1)
	Unknown	82 (1.3)
**Family status, n (weighted %)**		
	Married or in cohabitation	3936 (61.0)
	Single	1995 (38.0)
	Unknown or no answer	62 (1.0)
**Household Income, n (weighted %)**		
	Up to 900€	2401 (39.8)
	900€-1700€	1658 (28.1)
	>1700€	606 (10.9)
	No answer	1328 (21.3)
**Employment status, n (weighted %)**		
	Employed	2089 (38.7)
	Retired or household	2641 (35.4)
	Unemployed	784 (15.3)
	Other or unknown	479 (10.6)
**Country of birth, n (weighted %)**		
	Greece or Cyprus	5327 (87.4)
	Balkans	297 (5.9)
	East Europe or Former Soviet Union	89 (1.7)
	West Europe or Australia or America	101 (1.7)
	Africa	42 (0.9)
	Asia	51 (1.1)
	Unknown	86 (1.4)

## Discussion

### Strengths and Limitations

EMENO is one of the first population-based surveys focused on CVD and chronic respiratory diseases and their risk factors, which also includes anthropometric and physical examinations and blood tests as well as prevention measures uptake, health care facilities, and medicine use for the adult population in Greece. To the best of our knowledge, such data are rarely combined. The main goal or benefit of the EMENO study is that it provides valid estimates of various health indices. Study design and sampling procedure make EMENO one of the most representative of the current general adult population in Greece, thus enabling national authorities to develop tailored and more efficient strategies for disease prevention and management.

Expected benefits at local and international level are multifaceted. Greece will be able to provide European and international agencies with nationwide estimates of the prevalence of chronic diseases and health risks. Moreover, available data will help society with the necessary evidence to rationally monitor health systems and adjust health policies. Participants, local authorities, and nongovernmental organizations will be informed and become aware of health indices, potential inequalities, and impacts of modern lifestyles and behaviors. This increased awareness will contribute to successfully implement measures to resolve inequalities and to future lifestyle changes. Importantly, some participants have personally benefited already from the diagnosis of previously undiagnosed conditions.

As already mentioned, in EMENO, a blood specimens’ storage bank was established providing the opportunity for future studies to estimate the prevalence of other conditions with minimal additional cost. Having also obtained participants’ consent for DNA analyses makes EMENO a powerful resource to investigate this study’s and future hypotheses relating to environmental, lifestyle, biochemical, and genetic causes of CVD and chronic respiratory diseases in a representative Mediterranean population. EMENO has purposefully adopted harmonized data collection methods that allow data linkage with national or international cohorts or surveys. In case inequalities would be identified, EMENO results will generate new scientific hypotheses prompting to future-focused studies, for example, further studies in specific subpopulations and/or health conditions, explorations of specific diseases, or in specific regions, hazardous environmental exposures.

Study materials (including protocols and questionnaires) will contribute to European and international operators, universities, and research groups aiming to compare and harmonize health surveys and health indices, as the experience gained through studies’ implementation will be shared with other European groups conducting health surveys. Scientific reports and papers will be of major interest to other public health scientists.

Within the framework of EMENO, a network of specialists of different disciplines (epidemiologists, statisticians, internal medicine physicians, pneumonologists, microbiologists, public health experts, and database experts) as well as an official collaboration of all Greek schools of medicine has been established. Retaining such a network will be an added value and scientists’ mobility and interaction will be boosted. The steering committee will put all its effort to ensure network’s sustainability.

Following the successful example of NHANES study, studies such as EMENO could be repeated (possibly with a different focus) in a 5-year time to assess the temporal trends in health indicators and possibly to also investigate health conditions additional to those of the original study health conditions. If such a study would be approved and if adequate funds are ensured, study’s participants could be followed up (eg, through telephone interviews) to assess their health progression. The financial burden of such a study will be relatively low, whereas the added values (estimation of diseases’ incidence and of life expectancy) will be massive. In such a case, the study will be transformed from a cross-sectional to a cohort one.

Despite the significant benefits of EMENO, there are also some limitations. One major limitation was the sampling methodology and specifically the door-to-door approach for data collection. Although this method is quite common for conducting health screenings [32,33] and is the main approach used by the NHANES [34,35], Hillier et al [23] claimed that “the door-to-door method is too costly for researchers, too intrusive for participants and too dangerous for interviewers.” Conducting interviews face-to-face contribute to the quality of data collected but some serious considerations arise, mainly about interviewers’ safety. It demands a very well-experienced and trained staff; a good amount of time spent for their training and regular team meetings; however, on the other hand, it also allows for further social observations of the household and neighborhood conditions, which can add valuable information to the studies’ main purposes [36]. Another limitation was that for feasibility reasons, we only visited large islands (Rhodes, Corfu, and Lesbos), excluding small ones, which may be more likely to face problems to access to health services.

### Conclusion

The EMENO results will improve thus our understanding on health and health risks of people living in Greece, which will contribute to the evidence-based evaluation of health policies and preventive actions. Using EMENO results as background information, future health burden can be estimated for the main diseases under investigation (CVDs, chronic respiratory diseases, and diabetes). Predictions of future disease burden can be combined with collected data on the use of health services and medicines and with health economics models to estimate future health needs and costs. In conclusion, EMENO comprises a unique health data resource and a valuable bio-resource in a Mediterranean population.

## Appendix

Multimedia Appendix 1 EMENO Questionnaire Sources.

Multimedia Appendix 2 Standardized Instruments.

## Abbreviations

CAPI: computer-assisted personal interview
COPD: chronic pulmonary obstructive disease
CVD: cardiovascular disease
EMENO: National Survey of Morbidity and Risk Factors
HES: health examination surveys
ICF: informed consent form
NHANES: National Health and Nutrition Exam Survey
PSU: primary sampling unit
SC: scientific committee
SOP: standardized operating procedure
