# Supplementary material for: National Survey of Morbidity and Risk Factors (EMENO): Protocol for a Health Examination Survey Representative of the Adult Greek Population
Source: JMIR Res Protoc. 2019 Feb 4;8(2):e10997. doi: 10.2196/10997 (PMC6378546; doi:10.2196/10997)
Supplement: Multimedia Appendix 1 [file resprot_v8i2e10997_app1.pdf]

Appendix 1: EMENO Questionnaire Sources

National Survey of Morbidity and Risk Factors (EMENO)

References – EMENO Questionnaire

Information	Questionnaire Source
**Basic Information**	
Basic Household Information	EHES [1]
Basic Personal Information	EHES [1] – EMENO working group
**Health Status**	
General Health	EHES [1]
Anxiety and depressive symptoms	Patient Health Questionnaire – 4 (PHQ-4) [2]
Cardiovascular – Coronary Heart Disease	NHANES [3]
Cardiovascular – Peripheral Arterial Disease	[4, 5]
Respiratory	European Community Respiratory Health Survey (ECRHS) [6]
Sleep	EMENO working group
Well-being	Flourishing Scale [7]
**Health System**	
Health Services' Use	EMENO working group
Health Services' Satisfaction	EMENO working group
Health Care Costs	EMENO working group
Medicine – Food Supplements Usage	EMENO working group
Arterial Pressure Measurement	EHES [1]
Cholesterol Measurement	EHES [1]
Blood Glucose Measurement	EHES [1]
Screening for Cervical Cancer: Pap Smear Test	NHANES 2010 reduced
Screening for Breast Cancer: Mammography (analog or digital) – Breast MRI	NHANES 2010 reduced
Screening for Colorectal Cancer: Colonoscopy - Sigmoidoscopy	NHANES 2010 reduced
Screening for Colorectal Cancer: Fecal Occult Blood Test	NHANES 2010 reduced

Screening for Prostate Cancer: Prostate Specific Antigen (PSA)	NHANES 2010 reduced
Adult Vaccination for HPV	NHANES 2010 reduced
Adult Vaccination for Flu	NHANES 2010 reduced
**Factors Affecting Health**	
Height and Weight	EHES [1]
Physical Activity	IPAQ-short GR [8, 9]
Adherence to Mediterranean Diet	Mediterranean Diet Adherence Screening (MEDAS) Questionnaire [10]
Food Insecurity	Food Insecurity (3 items scale) [11]
Smoking	EMENO working group
Environment	EMENO working group
Alcohol Consumption	EMENO working group
Household Income	Deciles come from EU-Statistics on *Income* and Living Conditions [12]

References

1. Ahonen S, Koponen P, Tolonen H. EHES Manual: Fieldwork procedures, 2011. European Health Examination Survey. https://www.julkari.fi/bitstream/handle/10024/104392/URN_ISBN_978-952-245-843-8.pdf?sequence=1. Archived at: http://www.webcitation.org/6xZTyCLCJ). Accessed February 26, 2018
2. Kroenke K, Spitzer RL, Williams JB, Monahan PO, Lowe B. Anxiety disorders in primary care: prevalence, impairment, comorbidity, and detection. *Ann Intern Med*. 2007; 146(5): 317-25. PMID: 17339617
3. National Health and Nutrition Examination Survey. *Cardiovascular Disease* 2011-2012. https://www.cdc.gov/nchs/data/nhanes/nhanes_11_12/cdq.pdf. Archived at http://www.webcitation.org/6xZUlGSL7. Accessed February 26, 2018
4. Leng GC, Fowkes FG. The Edinburgh Claudication Questionnaire: an improved version of the WHO/Rose Questionnaire for use in epidemiological surveys*. J Clin Epidemiol.* 1992; 45(10): 1101-9. PMID: 1474406
5. Sever PS, Dahlöf B, Poulter NR, et al. Prevention of coronary and stroke events with atorvastatin in hypertensive patients who have average or lower-than-average cholesterol concentrations, in the Anglo-Scandinavian Cardiac Outcomes Trial - Anglo-Scandinavian Cardiac Outcomes Trial--Lipid Lowering Arm (ASCOT-LLA): a multicentre randomised controlled trial. *Lancet.* 2003; 361(9364): 1149-58. PMID: 12686036
6. European Community Respiratory Health Survey II (ECRHSII). *ECRHS II Screening Questionnaire*. 1998 15/01/2013]; Available from:

http://www.ecrhs.org/Quests.htm. Archived at: http://www.webcitation.org/6xaaTgf3p. Accessed February 26, 2018

7. Diener E, Wirtz D, Tov W, Kim-Prieto C, Choi D, Oishi S, Biswas-Diener R. New measures of well-being: Flourishing and positive and negative feelings. *Social Indicators Research.* 2009. 39: 247-266. DOI 10.1007/978-90-481-2354-4 12
8. Papathanasiou, G, Georgoudis G, Papandreou M, et al. Reliability measures of the short International Physical Activity Questionnaire (IPAQ) in Greek young adults*. Hellenic J Cardiol.* 2009. 50(4): 283-94. PMID: 19622498
9. Papathanasiou G, Georgoudis G, Georgakopoulos D, Katsouras C, Kalfakakou V, Evangelou A. Criterion-related validity of the short International Physical Activity Questionnaire against exercise capacity in young adults*. Eur J Cardiovasc Prev Rehabil.* 2010; 17(4): 380-6. PMID: 19940775
10. Hebestreit K, Yahiaoui-Doktor M, Engel C, et al. Validation of the German version of the Mediterranean Diet Adherence Screener (MEDAS) questionnaire. *BMC Cancer.* 2017, 17:341. PMID:28521737
11. Swindale A, Bilinsky P. Development of a universally applicable household food insecurity measurement tool: process, current status, and outstanding issues. *J Nutr*. 2006; 136(5): 1449S-1452S. PMID:16614442
12. EU-Statistics on *Income* and Living Conditions. 2011. http://ec.europa.eu/eurostat/web/microdata/european-union-statistics-on-income-and-living-conditions. Archived at: http://www.webcitation.org/6xaouubfu. Accessed February 26, 2018
