# Supplementary material for: National Survey of Morbidity and Risk Factors (EMENO): Protocol for a Health Examination Survey Representative of the Adult Greek Population
Source: JMIR Res Protoc. 2019 Feb 4;8(2):e10997. doi: 10.2196/10997 (PMC6378546; doi:10.2196/10997)
Supplement: Multimedia Appendix 2 [file resprot_v8i2e10997_app2.pdf]

Appendix 2: Standardized instruments

**Measurement**	**Instrument**
Height	Measuring Rod: TANITA HEIGHT ROD
Weight	Digital portable certified scale: SECA MOD 877
Arterial Blood Pressure	Semi-automated oscillometric devices Microlife Watch BP Home (Microlife AG, Widnau, Switzerland)
Respiratory Function	Spirometer: Spiropalm, Cosmed Srl, Italy
